# Modeling Alzheimer’s disease using human cell derived brain organoids and 3D models

**DOI:** 10.3389/fnins.2024.1434945

**Published:** 2024-08-01

**Authors:** Sarah Fernandes, Jasmin Revanna, Joshua Pratt, Nicholas Hayes, Maria C. Marchetto, Fred H. Gage

**Affiliations:** ^1^Laboratory of Genetics, The Salk Institute for Biological Studies, La Jolla, CA, United States; ^2^Department of Biological Sciences, University of California, San Diego, La Jolla, CA, United States; ^3^Department of Biology, San Diego State University, San Diego, CA, United States; ^4^Department of Biological Sciences, California State University, San Marcos, CA, United States; ^5^Department of Anthropology, Center for Academic Research and Training in Anthropogeny (CARTA), University of California, San Diego, La Jolla, CA, United States

**Keywords:** brain organoids, Alzheimer’s disease, aging, neurodegenerative diseases, APOE, amyloid beta, hyperphosphorylated tau, cerebral organoids

## Abstract

Age-related neurodegenerative diseases, like Alzheimer’s disease (AD), are challenging diseases for those affected with no cure and limited treatment options. Functional, human derived brain tissues that represent the diverse genetic background and cellular subtypes contributing to sporadic AD (sAD) are limited. Human stem cell derived brain organoids recapitulate some features of human brain cytoarchitecture and AD-like pathology, providing a tool for illuminating the relationship between AD pathology and neural cell dysregulation leading to cognitive decline. In this review, we explore current strategies for implementing brain organoids in the study of AD as well as the challenges associated with investigating age-related brain diseases using organoid models.

## 1 Introduction

Humans (*Homo sapiens*) are living longer than ever (Wheeler and Kim, 2011). While researchers are gaining insight into life prolonging interventions, we lack a mechanistic understanding of age associated brain diseases that diminish quality of life. Age-related brain diseases are challenging to model *in vitro* due to their complexity that includes contributions of multiple cell types, brain regions, and heterogenous pathological features, impacting both large-scale brain networks and local (small-world) cortical network organization responsible for high-level cognition (He et al., 2008; Sanz-Arigita et al., 2010; Sun et al., 2012). AD is one such age associated brain disease characterized by the accumulation of extracellular amyloid-β (Aβ) plaques and intracellular neurofibrillary tangles (NFTs), with NFTs demonstrating a stronger correlation to cognitive decline (Coedert et al., 1989; Bennett et al., 2004; Josephs et al., 2008; Moloney et al., 2021). It is unclear how these pathologies are triggered or what temporal roles they might play in neuroinflammation and disease progression (Akama and Van Eldik, 2000; Cribbs et al., 2012; Sudduth et al., 2013; Ahmad et al., 2019). Despite the greater association of NFTs with cognitive decline, cerebral spinal fluid (CSF) ratio of the 42-amino acid β-amyloid peptide (Aβ42) to the 40-amino acid β-amyloid peptide 40 (Aβ40) is a useful diagnostic tool of AD. Positron emission tomography (PET) has revealed that CSF Aβ42/40 ratio correlates better with AD pathology than Aβ42 alone (Lewczuk et al., 2014, 2017).

Familial AD (fAD) is an early onset form of AD caused by inherited genetic mutations in amyloid precursor protein gene (*APP*), presenilin 1 gene (*PSEN1*), or presenilin 2 gene (*PSEN2*) (Alzheimer’s Association, 2006). Unlike fAD, the more prevalent sAD form of AD cannot be ascribed to specific genetic mutations. Despite the multifactorial risks associated with developing sAD, it is greatly influenced by an abundance of inherited genetic risk variants that may act in a concerted manner (Katsumata et al., 2022). The *APOE* haplotype is one of the greatest influencing genetic risk factors for developing sAD, with the ε4 allele correlated with increased risk and the ε2 allele correlated with reduced risk (Michaelson, 2014; Yamazaki et al., 2019). In white individuals homozygous for *APOE4*, risk is increased by 15-fold and that risk increases to 33-fold for East Asian individuals. African Americans and Hispanic populations exhibit lower risk, ranging from 1- to 5-fold increases (Miyashita et al., 2023). Despite the known association between *APOE4* and AD, how the *APOE4* haplotype influences AD onset and progression or how *APOE2* provides protection is still unknown. Furthermore, multitudes of environmental factors have been reported to contribute to the etiology and pathogenesis of age-related diseases; a challenging paradigm to recapitulate *in vitro* (Fuso et al., 2005; Masoud et al., 2016; Vasanthakumar et al., 2020; Li et al., 2021; Smith et al., 2021). While aging is the greatest risk factor of developing age-related brain diseases like sAD (Qiu et al., 2009), whether aging causes sAD is debatable and beyond the scope of this review. However, it has been suggested that a gene or set of genes with a type of pleiotropy which confers beneficial or neutral effects in an individual during youth could have deleterious effects in the context of aging and, as a result, has evaded natural selection (Williams, 1957; Wheeler and Kim, 2011).

Rodent models of AD have aided in the understanding of AD etiology and pathogenesis, albeit rodents do not inherently develop Aβ plaques or NFTs (Götz et al., 2018). The AD pathology observed in the majority of rodent models is induced via mutations in genes associated with fAD, including *APP* or *PSEN1*, which account for only about 5% of all AD cases (Alzheimer’s Association, 2006). Mouse (*Mus musculus*) models exhibiting hyperphosphorylated tau in the somatodendritic domain have been established by expressing a mutation in familial FTLD *MAPT* under the control of the human promoter *THY1.2* (Götz et al., 1995). More precise gene editing has been employed to humanize rodent models and achieve endogenous expression levels, yet these models often fail to recapitulate human specific responses to the induced pathological features, such as those by human microglia or the neuroinflammatory response (Sasaguri et al., 2017; Götz et al., 2018). While stereotaxic injections of transgenic rodent or human AD brain lysates revealed complex amplification of proteopathic aggregates in mice, it is difficult to untangle the effects of the invasive injections and what could be the specific response of the mouse model (Bolmont et al., 2007; He et al., 2018). Rodent models cannot replicate the genetic diversity of humans that may contribute to the overall risk of developing sAD (Götz et al., 2018). Behavioral differences between humans and rodents might also limit success of preclinical trials (Salloway et al., 2014). Additionally, rodents are not a long-lived species and require genetic manipulation to mimic human susceptibility to age-related disease (Harkema et al., 2016; Cabral et al., 2021). Nonhuman primates, like rhesus macaques (*Macaca mulatta*), could bridge the gap in lifespan between rodents and humans; however, these studies would be long, costly, and have high ethical considerations (Kennedy et al., 2014). Rhesus macaques demonstrate spontaneous cerebral amyloidosis upon aging with features similar to human plaques, yet the age of onset and distribution of plaques could have important differences, plus observed tauopathies are minimal (Van Dam and De Deyn, 2017). Nonhuman animal models of AD are necessary for relating behavioral and cognitive changes to pathological features and in understanding safety of potential therapeutics; however, model systems that better recapitulate the human specific biology related to AD are required.

The advent of techniques for the reprogramming of donor somatic cells to human induced pluripotent stem cells (hiPSCs) and the subsequent differentiation of iPSCs to neural cell types, has provided researchers access to human specific cellular processes of brain development and maturation (Takahashi et al., 2007). hiPSCs maintain the genetic background of the donor from which they are derived while overwriting most of the epigenetic signatures an individual has acquired throughout one’s life (Marchetto et al., 2009; Hu et al., 2010; Kim et al., 2010; Shutova et al., 2016). This might allow for the disentanglement of genetic versus epigenetic influences in the context of age-related brain disease. hiPSCs can be employed to generate brain organoids that recapitulate some features of brain development in three-dimensional (3D) structures consisting of various subtypes of neuronal and glial cell populations (Lancaster and Knoblich, 2014; Matsui et al., 2018). These 3D models have increased tissue architecture when compared to 2D monolayer culture and both inhibitory and excitatory synapses that give rise to spontaneous neuronal activity suggesting functional maturity of neurons (Lancaster et al., 2013; Quadrato et al., 2017; Trujillo et al., 2019a). Although brain organoids are currently limited in the amount of maturation that can be achieved, and sAD is a late onset disease, brain organoids could demonstrate sAD phenotypical differences apparent in neurodevelopment, exhibit greater cellular maturation and complexity compared to 2D monolayer cell culture, or represent physiological responses to aging simulating cellular stresses (Chiaradia and Lancaster, 2020). These features of brain organoids allow for the investigation of sAD pathogenesis in a human specific model and could accelerate the development of therapeutic or preventative strategies in sAD.

Most protocols for brain organoid derivation lack microglia since microglia develop from the mesodermal-derived yolk-sac and migrate into the brain during early development while other brain cell types comprising brain organoids originate from ectoderm (Ginhoux et al., 2010; Utz et al., 2020). Coculturing brain organoids with hiPSC or human embryonic stem cell (ESC) derived microglia, which are major cellular players in the etiology and pathogenesis of age-related brain diseases including AD, could untangle microglia influences on, or responses to, AD pathology (Plescher et al., 2018). Microglia can be integrated into donor matched brain organoids allowing for the study of donor-specific genetic influence on microglia interactions during brain maturation (Schafer et al., 2023). Throughout this review, we will consider whether brain organoids of increasing complexity could serve as models of age-related brain diseases either by representing developmental neurobiology that might foreshadow age-related brain diseases or via the perturbation of specific mechanisms, pathways, or homeostatic processes to induce age-related brain disease pathologies in organoids. We will discuss the application and challenges of brain organoids enriched with astrocytes, microglia, and oligodendrocytes to unravel cell-to-cell interactions and genetic contributions in age-related brain disorders with a focus on AD.

## 2 Generating brain organoid models of AD

**Figure 1:**
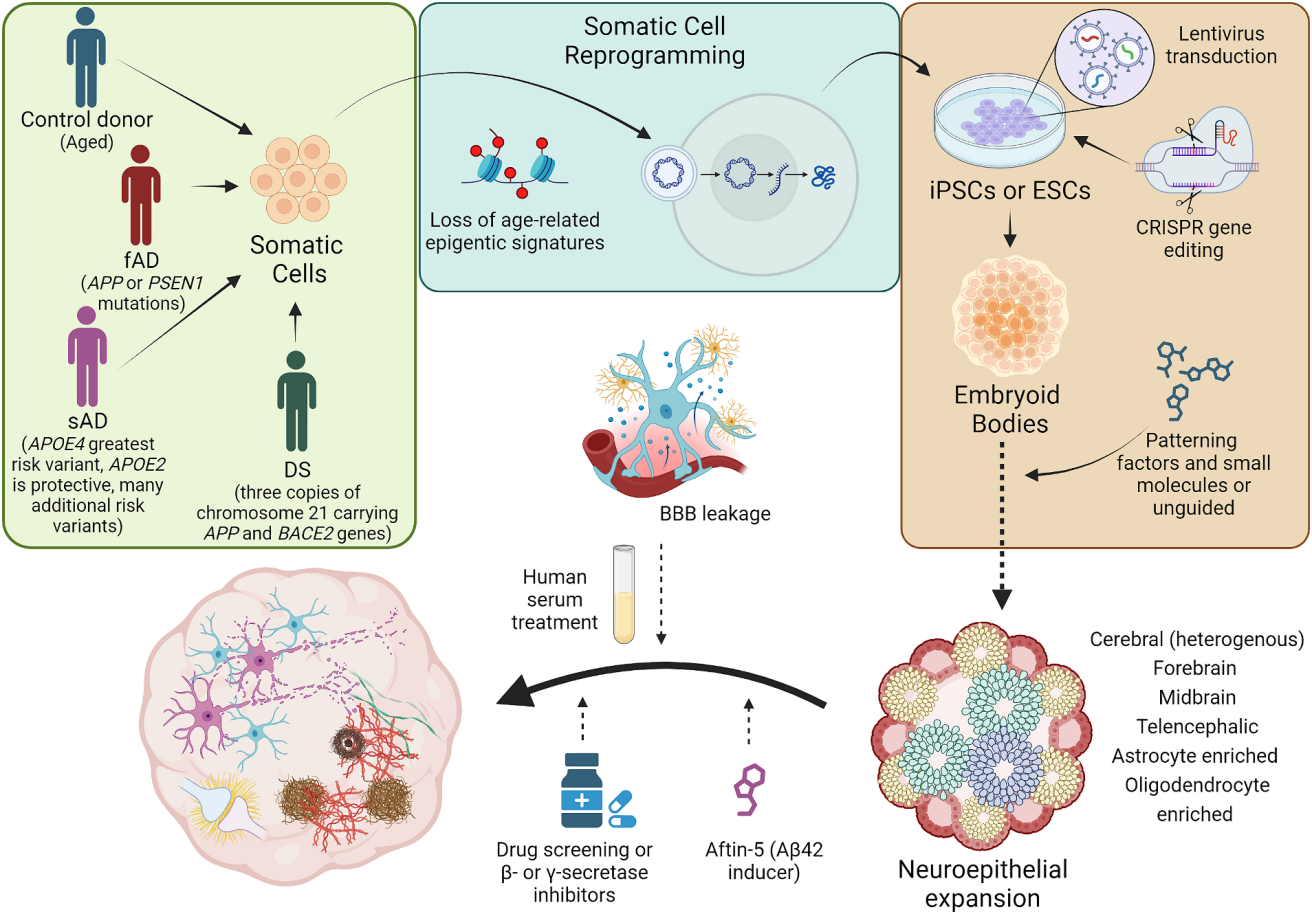
Schematic diagram illustrating the generation of brain organoid models of AD. Brain organoids generated from patient or control donor somatic cells will retain the genetic background of the patient or donor. Most, if not all, age-related epigenetic signatures from the donor will be lost during the reprogramming of somatic cells to iPSCs. Genetic modifications associated with AD can be introduced into iPSCs or ESCs using techniques including lentivirus overexpression or CRISPR induced point mutations and gene knockouts. Genome edited PSCs can then be employed in the generation of embryoid bodies which could be patterned to obtain more directed, brain region specific organoids or unguided approaches can be implemented to yield more heterogeneous cerebral organoids. Following organoid cellular maturation, brain organoids could be treated with factors to simulate age-related events like BBB leakage, to induce AD pathology, to observe the effects of potential pharmaceuticals on AD pathology, or to inhibit mechanisms hypothesized to effect AD pathogenesis. These strategies have successfully established brain organoids with some AD pathological features, including Aβ aggregates, NFTs, increased apoptosis, and neuronal network dysregulation. fAD, familial Alzheimer’s disease; sAD, sporadic Alzheimer’s disease; DS, Down syndrome; iPSCs, induced pluripotent stem cells; ESCs, embryonic stem cells; Aβ, amyloid-β; BBB, blood brain barrier. Not to scale.

Limited access to developing and diseased human brain tissues has motivated researchers to pursue *in vitro* models, providing a window into concealed processes of brain development and pathogenesis. Following the isolation of hESCs and establishment of successful hESC culture conditions (Thomson et al., 2013), floating embryoid bodies (EBs) of aggregated hESCs were generated and plated to obtain neurons (Bain et al., 1995). Lancaster et al. (2013) took the next step to generate cerebral organoids by embedding EBs in Matrigel and supporting a suspension culture in media with minimal influence on cell lineage specification. Cerebral organoids demonstrate ventricular zone (VZ) and subventricular zone (SVZ)-like layering with differentiating neurons that migrate outwards to form heterogenous structures with forebrain, midbrain, and hindbrain identities (Lancaster et al., 2013; Lancaster and Knoblich, 2014). More recently, factors and small molecules have been incorporated into brain organoid generation protocols to direct cell fate and regional identity of brain organoids. Often guided approaches begin with dual SMAD inhibition (the inhibition of both transforming growth factor beta (TGF-β) and bone morphogenetic protein (BMP) pathways) to yield robust ectoderm (Chavali et al., 2020), followed by dorsalizing factors to promote forebrain identity (Liem et al., 1995; Qian et al., 2018), or exposure to WNTs and BMPs for dorsomedial identities including pallial hem with hippocampus (Sakaguchi et al., 2015). Brain regions including the hippocampus, entorhinal cortex (EC), primary visual cortex, superior frontal gyrus (SFG), and middle temporal gyrus are histopathologically relevant to AD (Liang et al., 2008). Although brain organoids do not result in the six distinct layers of the human cortex, discrete subregions of the adult cortex, or the complex cellular organization of the hippocampus, brain organoids that are more cortical or hippocampal in derivation could better represent cell types implicated in AD (Chen et al., 2021). Brain organoids model some early cytoarchitectural features of brain development, including organization of SVZ and VZ-like layering, until approximately 4 months. Beyond 4 months of generation, brain organoids continue to recapitulate some developmental transcriptional programs, however SVZ and VZ-like layering begins to dissipate (Camp et al., 2015; Tanaka et al., 2020; Uzquiano et al., 2022). Nevertheless, amyloid-β (Aβ) aggregates and NFTs have been observed in brain organoids derived from fAD donor hiPSCs (Raja et al., 2016; Gonzalez et al., 2018; Park et al., 2021). The complex extracellular environment of brain organoids could be conducive for Aβ protein aggregation (Chiaradia and Lancaster, 2020). Additionally, the lack of vasculature in brain organoids likely prevents physiological clearance of Aβ which would occur through the blood brain barrier (BBB), and the lack of microglia results in decreased uptake and degradation of Aβ (Qi and Ma, 2017). However, a key question remains outstanding; can brain organoids serve as models of AD and other age-related brain diseases? Here, we discuss strategies and challenges in elucidating age-related brain diseases using brain organoids (Table 1; Figure 1).

**Table 1:** Overview of primary literature using brain organoids to investigate AD and AD-related pathological features.

**Model system**	**Description of model including induced pathology/genetic background/mutated gene(s)**	**Age of organoids at readout**	**Findings**	**Rescue**	**Rescue phenotype**	**Reference**
**iPSC-derived cerebral organoids or 3D neuronal spheroids**	*iPSCs patient-derived cohort D-CAA (APP point mutation).*	~ 4 months (110 days) and ~ 2 months (52 days)	Increased neuronal differentiation and maturation potential in D-CAA. Increased transcription of *vGLUT1*, *TGFBR1*, *AAQP4*, and *GFAP* in D-CAA. Increased Aβ (4G8) deposition in D-CAA. No significant difference in p-tau.	CRISPR-edited isogenic	NA (Variability between biological triplicates warranted more isogenic lines)	Daoutsali et al. (2023)
	*iPSCs donor cohort (no diagnosis of dementia) APOE-ε3/ε3 and APOE- ε4/ε4.* *CRISPR-edited isogeneic APOE-ε4/ε4 to APOE—ε3/ε3.*	6 months	No significant differences in APOE, Aβ42, Aβ40, or Aβ42/Aβ40 ratio, p-tau (S396 and T231) protein expression that could be attributed to *APOE* status	Isogenic *APOE-ε3/ ε3*	Decrease in p-tau S199	Hernández et al. (2022)
	iPSCs donor cohort *APOE-ε3/ε3 and APOE- ε4/ε4 from control donors or patients diagnosed with sAD.* *CRISPR-edited isogeneic APOE-ε4/ε4 from sAD patient to APOE—ε3/ε3.*	1, 2, and 3 months	Increased cleaved CASP3 in AD-E4 and decreased synaptophysin and PSD95 in AD at 12 weeks. Increased organoid maturation in AD around 4 weeks. Increased Aβ42, Aβ40, and Aβ42/Aβ40 in AD at 12 weeks. Increased p-tau (AT8) and soluble apoE in Con-E4, AD-E3, and AD-E4. Enrichment of gene sets involved in RNA metabolisms affected in AD	Isogenic *APOE-ε3/ ε3*	Reduction of cleaved CASP3, Aβ42, Aβ40, p-tau, and G3BP (no change in Aβ42/Aβ40) at 12 weeks	Zhao et al. (2020)
	iPSC-derived 3D neural models embedded in Matrigel-coated alginate capsules and cultured with BrainPhys media	25 weeks (~6.25 months or 175 days)	Expression of all 6 adult isoforms of tau (5 isoforms of tau were detected at week 25 in 3D models cultured without BrainPhys). Proportion of isoforms did not match that found in the adult human brain.	NA	NA	Miguel et al. (2019)
	iPSCs donor cohort fAD, DS, CJD, and healthy controls.	~ 4 months (110 days)	Increased Aβ deposits, Aβ42, Aβ40, Aβ42/Aβ40, p-tau (AT8 and PHF-1), and NFT structures in fAD and DS. Increased CASP3 in DS.	NA	NA	Gonzalez et al. (2018)
	3D neuronal spheroids - Patient cohort iPSCs from 5 patients with sAD diagnosis (some had PET scans).	~ 2 months (63 days)	sAD spheroids showed Aβ and p-tau (Thr181).	BACE1 and γ-secretase inhibitors.	Decreased Aβ40 and Aβ42. Clathrin and APP were reduced in BACE1 inhibitor responsive spheroids compared to a less responsive line. Neuronal spheroids showed less of a reduction in Aβ compared to 2D neurons derived from the sAD patients.	Lee H. K. et al. (2016)
**Gene edited cerebral organoids or 3D embedded cell suspension**	iPSCs donor cohort DS (T21), isogenic iPSC clones with disomy 21 (D21) from same DS individual, non-DS individual with triplication of *APP* gene causing fAD (Dup*APP*), CRISPR eliminated *BACE2* in T21 (Δ7).	~ 3 to 4 months (100 to 137 days)	Increased Aβ19, Aβ20, and Aβ34 (Aβ degradation and preventing products) in T21. Decreased Aβ19, Aβ20, and Aβ34 and increased amyloid deposits and tau pathology (AT8 and TG3) in Δ7.	Β- and γ-secretase inhibitors	Complete loss of amyloid deposits and decreased number of neurons expressing pathologically conformed tau (TG3) in Δ7.	Alić et al. (2021)
	CRISPR *PITRM1* knockout iPSCs, from a non-affected control donor, (PITRM1^-/-^) derived cerebral organoids.	1, 2, and 6 months	scRNA-seq revealed dysregulated mitochondrial function, oxidative phosphorylation, synaptogenesis, long-term potentiation, inflammatory pathways, and sirtuin in PITRM1^-/-^. Increased APP, Aβ40, Aβ42, and Aβ42/Aβ40 ratio in PITRM1^-/-^ at 2 months. Increased CASP3 positive cells at in in PITRM1^-/-^ starting at 2 months but no increased cell death was observed at later time points.	NAD+ precursor, NMN (enhances mitochondrial clearance)	Decreased Aβ42/Aβ40 ratio, p-tau/total tau ratio, and the number of CASP3 positive cells in PITRM1^-/-^ organoids.	Pérez et al. (2021)
	3D embedded cell suspension - ReN cells with *APOE-ε3/ε3 genotype* embedded in Matrigel. Lentiviral constructs used to overexpress *APP* or *APP* and *PSEN1* (fAD mutations) in ReN cells (fAD ReN cells).	~ 1 month (42 days)	Increase in the levels of 4R adult tau isoforms in 3D compared to 2D. Increased Aβ42, Aβ40, Aβ42/Aβ40 ratio, amyloid deposits, and p-tau (Ser199, Ser202, Thr205, and AT8) in fAD ReN cells.	ꞵ- and γ- secretase inhibitors. GSK3β inhibitors 1-azakenpaullone (1-AZA) and SB415286 (SB41).	β- and γ- secretase inhibitors decreased Aβ deposits, p-tau-positive cell numbers, and p-tau levels in neurites in fAD ReN cells. 1-AZA and SB41 decreased p-tau in fAD, APP and PSEN1 overexpressed ReN cells.	Choi et al., 2014
**Exogenous induction of AD-like pathology in brain organoids**	iPSC-derived brain organoids treated with 10% human serum for ~12 days.	~ 3 months (90 to 110 days)	Increased Aβ, Aβ aggregates, BACE protein, p-tau (Thr181), and phosphorylated/total GSK3α/β ratio after serum treatment. Decreased SYN1^+^ synaptic puncta, calcium signaling, number of spikes, bursts, mean firing rate, and synchrony index after serum treatment.	BACE inhibitor IV (IV), GSK3α/β inhibitor CHIR99021 (CHIR)	IV reduced Aβ under serum exposure but not p-tau levels. CHIR reduced phosphorylated and total GSK3α/β and p-tau with serum exposure but not Aβ levels. Neither IV or CHIR increased SYN1^+^ synaptic puncta.	Chen et al. (2021)
	iPSC-derived brain organoids treated with Aβ42 inducer, Aftin-5.	1, 2, and 3 months	Increased Aβ42/Aβ40 ratio with Aftin-5 treatment. Increased APP and prion protein (PrP^C^) between 1 and 3 months.	NA	NA	Pavoni et al. (2018)
Network based, cerebral organoid drug screening platform	PiB-negative or PiB-positive (sAD) donor derived iPSCs used to generate cerebral organoids (iCOs). CRISPR edited APOE4 isogenic (E4^iso^) iCOs (E3 parental line).	2 months (60 days)	PiB^+^ iCOs secreted higher Aβ42, Aβ40, total tau, and p-tau with APOE4 carriers and E4^iso^ iCOs secreting increased levels. PiB^+^ and E4^iso^ iCOs had increased calcium peaks and downregulated genes related to synaptic function and neurogenesis.	Silico perturbation analyses identified several drug candidates for targeted AD pathology like Fli and Ripa.	All identified candidate drugs reduced Aβ or tau deposition and enhanced or maintained cell viability to some extent.	Park et al. (2021)

The list of model systems is based on findings and is not exhaustive. iPSC, induced pluripotent stem cells; AD, Alzheimer’s disease; fAD, familial Alzheimer’s diseases; sAD, sporadic Alzheimer’s disease; D-CAA, Dutch-type cerebral amyloid angiopathy; DS, Down syndrome; CJD, Creutzfeldt-Jakob disease; ReN cells, ReNcell VM human neural precursor cells; NMN, nicotinamide mononucleotide; GSK3α/β, glycogen synthase kinase 3α/β; SYN1, synapsin-1; PiB, Pittsburg compound B; Fli, Flibanserin; Ripa, Repasudil.

### 2.1 Stem cell derived brain organoid models of sAD, fAD, and down syndrome related AD

Brain organoids are derived from either ESCs, isolated from the inner cell mass of a blastocyst (Thomson et al., 2013), or iPSCs that are obtained through the reprogramming of donor somatic cells (Takahashi et al., 2007). iPSCs experience a resetting of epigenetic and age-related signatures during the reprogramming process, providing iPSCs a pluripotency comparable to ESCs (Shutova et al., 2016). However, it has been suggested that some of the epigenetic and age-related signatures of the somatic cells from which iPSCs are reprogrammed could be retained and influencing iPSC fate specification during differentiation (Marchetto et al., 2009; Hu et al., 2010; Kim et al., 2010). If retention of somatic cell epigenetic signatures does occur during iPSC reprogramming, it could account for a portion of the high variability that has been observed in cerebral organoid models derived from different iPSC lines and challenges the ability to separate genetic versus epigenetic influence in AD. Hernández et al. (2022) developed cerebral organoids from the iPSCs of individuals with no diagnosis of dementia yet with *APOE-ε3/ε3* or *APOE-ε4/ε4* variants and from a clustered regularly interspaced short palindromic repeats (CRISPR)-Cas edited isogenic iPSC line of *APOE-ε4/ε4* to *APOE-ε3/ε3* to investigate the effects of *APOE* status related to late onset AD pathology. The researchers found no APOE dependent differences in APOE protein expression and amyloid-β secretion. Additionally, variations in phosphorylated tau (p-tau) could not be attributed to specific genotypes with the exception of a decrease in the phosphorylation of tau at S199 which was decreased in the isogeneic *APOE-ε3/ε3* organoids when compared to APOE-ε4/ε4(Hernández et al., 2022). Conceivably, combinatorial effects of APOE status and additional sAD associated genetic variants influence ones’ risk of developing sAD and accounts for the lack of effects observed (Lin et al., 2018). Zhao et al. (2020) provided evidence supporting the hypothesis that *APOE4* contributes to sAD onset through a combinatorial effect with other sAD genetic risk variants. Similarly, Zhao et al. (2020) generated cerebral organoids using iPSC lines from *APOE-ε3/ε3* or *APOE-ε4/ε4* carriers, however five of the hiPSC lines were from cognitively unimpaired individuals carrying *APOE-ε3/ε3*, five were from cognitively unimpaired individuals carrying *APOE-ε4/ε4*, five were from sAD patients carrying *APOE-ε3/ε3* (AD-E3), and five were from sAD patients carrying *APOE-ε4/ε4* (AD-E4). The researchers found increased cleaved Caspase-3 (CASP3), indicating increased apoptosis, and decreased synaptophysin and PSD95, suggesting synaptic loss, in AD-E4 cerebral organoids at week 12 revealing a synergistic APOE4 and sAD status effect. Corroborating observations made by Hernández et al. (2022) and Zhao et al. (2020) found that Aβ40, Aβ42, and Aβ42/Aβ40 ratio were increased in sAD organoids independent of *APOE4* status. Lastly, the researchers found that both *APOE4* and AD status increase p-tau levels in cerebral organoids, although *APOE4* had a significant effect on p-tau in both AD and healthy derived organoids (Zhao et al., 2020). This research highlights the importance of considering the broad range of genetic backgrounds contributing to an individual’s risk of developing sAD when establishing physiologically relevant, human models of sAD.

It is estimated that approximately 50% of people with Down syndrome (DS) will develop AD as they age. This is caused by an extra copy of *APP* that people with DS carry on a third copy of chromosome 21. Gonsalez and colleagues performed experiments to determine whether fAD or DS patient derived cerebral organoids develop AD pathology when compared to control donors or Creutzfeldt-Jakob disease (CJD) derived cerebral organoids. CJD is thought to be caused by an abnormal isoform of a prion protein. The authors generated cerebral organoids from the hiPSCs of a patient with fAD carrying a mutation in *PSEN1*, a 5-month-old patient with DS, a 66-year-old control donor, and two patients with CJD. After analyzing 50 to 200 cerebral organoids at day 110, they found Aβ deposits in fAD and DS derived cerebral organoids and no Aβ deposits in control donor or CJD derived cerebral organoids. Enzyme-linked immunosorbent assay (ELISA) revealed an increased Aβ42/Aβ40 ratio in both fAD and DS donor derived cerebral organoids when compared to control donor derived cerebral organoids. Moreover, NFTs and increased CASP3 were observed in fAD and DS derived cerebral organoids relative to control donor derived cerebral organoids (Gonzalez et al., 2018). An additional study developed cerebral organoids from a donor carrying a point mutation in APP causing Dutch-type cerebral amyloid angiopathy (D-CAA). No increase in the production of Aβ42 or Aβ40 in D-CAA iPSC derived neurons was found, however researchers observed an increase in Aβ deposition in D-CAA derived organoids relative to controls at both days 52 and 110, as well as an increase in transforming growth factor beta receptor 1 (TGFBR1), thought to promote APP and Aβ expression in astrocytes (Daoutsali et al., 2023). The fact that only approximately 70% of individuals with DS develop dementia by age 60 has led researchers to hypothesize that additional genes located on chromosome 21 could be influencing the onset and progression of age-related dementia. Β-site APP cleaving enzyme 2 (BACE2), a homolog of BACE1, is located on chromosome 21 and could have anti-amyloidogenic properties. When researchers used CRISPR-Cas9 to eliminate a copy of BACE2 (from three copies of BACE2 to one copy) in DS derived cerebral organoids, they saw a significant increase in Aβ plaque-like deposits by day 48 when compared to unedited DS derived cerebral organoids. Treatment of edited DS derived cerebral organoids with β and γ secretase inhibitors eliminated Aβ plaque-like deposits rescuing the observed effects (Alić et al., 2021). These studies suggest that brain organoid models derived from DS patients are useful in elucidating mechanisms of APP processing related to Aβ-deposition, however they might not represent AD associated tau pathology or more complex interactions of genetic variants contributing to sAD pathogenesis.

### 2.2 Genetic modifications to investigate fAD and mitochondrial dysfunction

One of the earliest 3D models of AD pathology was described by Choi et al., although this model is unlike stem cell derived brain organoids in that it employs ReNcell VM human neural precursor (ReN) cells that are embedded as a cell suspension into Matrigel and differentiated into neurons with projections yet minimal tissue architecture. Using lentiviral constructs, the ReN cells were transduced to overexpress human *APP* or *APP* and *PSEN1*, containing fAD mutations. fAD ReN cells showed extracellular amyloid-β deposits that were reduced upon treatment with β- or γ-secretase inhibitors. p-tau was increased in the neurites and neuronal cell bodies and, again, decreased following β- or γ-secretase inhibitors suggesting that increased APP processing resulted in elevated p-tau mislocalization (Choi et al., 2014). Although, in sAD, iPSC derived cortical spheroid models, β- or γ-secretase inhibitors were less effective at reducing Aβ42 levels when compared to iPSC derived, monolayer neural cells (Lee H. K. et al., 2016). While a similar method to that used by Choi et al. (2014) could be applied to generate organoids from stem cells that overexpress fAD mutations, it is unclear if organoids generated from sAD iPSCs would recapitulate pathology to the same extent. However, a recent study of thousands of individuals found that almost all APOE4 homozygous carriers, the biggest genetic risk factor for sAD, exhibited high levels of Aβ42 in CSF (Fortea et al., 2024). This supports the idea that fAD and sAD organoids could express AD pathology, and that investigating the cascade following Aβ accumulation is important in understanding AD pathogenesis.

More recently, researchers have investigated the effects of a missense loss of function mutation in pitrilysin metallopeptidase 1 (*PITR1*) causing mitochondrial dysfunction and an age-dependent, neurological syndrome with AD-like amyloidogenesis (Langer et al., 2018). Using CRISPR-Cas9, the *PITRM1* gene of hiPSCs obtained from an 80-year-old, control donor was targeted to introduce knockout mutations. Cerebral organoids were generated from *PITR1*^-/-^ iPSCs. At 2 months, western blot and immunohistochemistry (IHC) analyses of *PITR1*^-/-^ organoids revealed increased APP levels and tau hyperphosphorylation when compared to *PITR1*^+/+^ organoids. Additionally, IHC demonstrated increased CASP3 while immunoassays showed higher Aβ40, Aβ42, and Aβ42/Aβ40 ratio in *PITR1*^-/-^ organoids relative to controls. To determine whether enhanced mitochondrial mitophagy would decrease the observed AD pathology, *PITR1*^+/+^ and *PITR1*^-/-^ organoids were treated with the NAD+ precursor, nicotinamide mononucleotide (NMN). The researchers observed decreased Aβ42/Aβ40 ratio and p-tau/total tau ratio in addition to decreased CASP3 in *PITR1*^-/-^ organoids following treatment with NMN (Pérez et al., 2021). Further research applying gene editing tools, like CRISPR-Cas, to introduce either genetic or epigenetic modification associated with fAD or accelerated aging diseases could assist in elucidating specific processes associated with aging and AD. Nevertheless, it will be challenging to represent the genetic complexity of age-related brain diseases like sAD with gene editing alone.

### 2.3 Exogenous induction of AD-like pathology and events of aging

Researchers have worked to overcome the challenges associated with phenotypically young characteristics of human brain organoids by exogenously introducing events to induce late-onset, disease-like pathology. To mimic BBB leakage, an effect that often occurs with aging and at increased rates in individuals harboring *APOE4* variants, Chen et al. (2021) treated brain organoids derived from hiPSCs with 10 percent human serum for 12 days. Control brain organoids were not treated with serum, and brain organoids were analyzed between days 90 and 110. The authors observed increased insoluble Aβ and increased extracellular Aβ aggregates in serum-treated brain organoids when compared to controls. BACE, an enzyme that drives the production of Aβ, was also elevated in serum-treated brain organoids, and treatment of organoids with BACE inhibitor IV reduced Aβ levels in organoids even with serum exposure. Furthermore, the researchers observed synaptic loss measured by a decrease in synapsin-1 puncta, decreased calcium signaling identified with calcium imaging, and reduced neural network activity in the form of decreased number of spikes, bursts, mean firing rate, and synchrony index as measured by multi-electrode arrays (MEA) in brain organoids following serum exposure (Chen et al., 2021). This research supports the hypothesis that exogenous factors could recapitulate events associated with aging and induce age-related brain disease-like pathology in brain organoid models. However, serum is heterogenous, containing various factors that could influence cell behavior, and differences in the composition of serum components across individuals could affect results. Determining which components are contributing to observed pathological effects is challenging when using complex substances to evoke disease pathogenesis.

A more precise approach to induce age-related disease pathology is the use of small molecules or factors to modulate cellular pathways implicated in the pathological feature of interest. Pavoni et al. (2018) targeted the production of Aβ42 in brain organoids generated from control donor derived iPSCs using a small molecule inducer of Aβ42 called Aftin-5. The authors observed an increase in Aβ42 and Aβ42/Aβ40 ratio, however there was no increase in p-tau or extracellular amyloid-β as indicated by IHC (Pavoni et al., 2018). While the implementation of small molecules to induce AD pathology can teach us about the pathways related to the targeted pathological feature, potentially dysregulated cellular responses associated with a disease state would not be represented in a control donor derived brain organoid. It would be informative to treat sAD patient derived and control donor derived brain organoids with AD pathology inducers like Aftin-5 to observe cellular responses with patient specific genetic backgrounds. Exogenous treatment of AD derived brain organoids with synthetic Aβ40 and Aβ42 could provide further insight into multicellular responses to Aβ pathology. Primary brain cell and stem cell derived neural cell types treated with Aβ showed increases in endogenous Aβ release resulting in neuronal dysfunction. Aβ is thought to cause neuronal dysregulation through the activation of receptors associated with neuroinflammation, increased oxidative stress, loss of synapses, and a dysregulation of neurotransmitters (De Felice et al., 2007; Brito-Moreira et al., 2011; Ferreira et al., 2014; Izzo et al., 2014). The variety of cell types with complex interactions available in brain organoids may reveal cascading AD pathogenesis upon Aβ oligomer treatment (Fontana et al., 2020).

### 2.4 Limitations of necrosis and understanding the capacity for maturation

The lack of vascularization in brain organoids prevents sufficient diffusion of oxygen beyond the initial approximately 600 μm depth and restricts nutrient penetration, leading some researchers to limit their analysis to a region of neuron-rich, CASP3-sparse tissues about 250 μm from the organoid surface (Raja et al., 2016; Magliaro et al., 2019). Often, beyond the 250 μm depth into brain organoids, tissues abundant in apoptotic CASP3 are comprised of dead and dying cells with little to no protein expression forming a necrotic core. The restricted oxygen and nutrient diffusion limit is thought to inhibit cellular maturation, cellular subtype specification, and continued growth of brain organoids (Bhaduri et al., 2020; Chiaradia and Lancaster, 2020). Reports comparing single-cell RNA sequencing (scRNAseq) from developing human cortex and brain organoids found an increase in glycolysis and endoplasmic reticulum (ER) stress genes within brain organoids. The authors observed decreased cellular stress markers and increased cell subtype specification following transplantation of cells from dissociated, two-month-old organoids into mice suggesting that *in vitro* cellular stress was responsible for impairments in cellular subtype specification (Bhaduri et al., 2020). ER stress leads to the unfolded protein response which occurs during early stages of tauopathies and is strongly correlated with p-tau accumulation in AD. Human neuroblastoma, SK-N-SH, cells treated with an inhibitor of glucose metabolism (2-deoxy glucose) demonstrated increased p-tau, although p-tau levels returned to prior levels upon removal and washout of the drug (Van Der Harg et al., 2014). Furthermore, studies in mice suggest that metabolic stress associated oxidative stress increases brain inflammation and neuronal insulin resistance leading to Aꞵ deposition (Hahm et al., 2020). The evidence linking elevated cellular stress with AD pathology, in addition to the increased cellular stress observed in brain organoids, can complicate the interpretation of observed Aꞵ and p-tau changes in these models. This highlights the importance of including relevant controls and minimizing cellular stress when modeling AD using brain organoids.

Early strategies to overcome the deficiencies attributed to a lack of vasculature in brain organoid culture include maintaining organoids in spinning bioreactors and the addition of neurotrophic factors, like brain derived neurotrophic factor (BDNF) or glial derived neurotrophic factor (GDNF), to support neuronal maturation and survival (Lancaster et al., 2013; Quadrato et al., 2017; Qian et al., 2018). Additionally, slicing organoids to establish a sliced neocortical organoid (SNO) model reduces the thickness of the organoid structure and increases access of cells to nutrients and oxygen. The SNO model has demonstrated improved neuronal survival and cortical layer formation, although it is unclear if the regular slicing of organoids could introduce cellular stress (Qian et al., 2020). Implementing organoid generation protocols which promote more physiologically relevant proportions of microglia, astrocytes, and oligodendrocytes could better support the survival, maturation, and cortical layering of neurons during long term culture, nonetheless this will not improve oxygen diffusion limits within brain organoids (Madhavan et al., 2018; Cakir et al., 2022; Huang et al., 2022). Perhaps the most advanced brain organoid cellular maturation and complete compilation of cell types has been achieved through the transplantation of brain organoids into immunodeficient mice which increases oxygen and nutrient delivery into the core as the mouse vasculature infiltrates the transplanted organoid (Mansour et al., 2018; Wang et al., 2024). However, in the realm of disease modeling, it is difficult to untangle the influence of the rodent *in vivo* systems on the pathological features of the transplanted brain organoid.

Brain organoids are derived from embryonic-like cells and recapitulate several early fetal brain developmental programs across both guided and unguided brain organoid generation protocols (Tanaka et al., 2020). Understanding the extent of cellular maturation and specification that can be achieved during long-term culture of brain organoids is essential in elucidating their capacity to model late-onset and age-related brain diseases like AD. A recent study identified subpopulations of cells in brain organoids that might deviate from human fetal cortex transcriptional paradigms. This included a more gradual decline in cell cycle, neural stem cell (NSC), and neural progenitor cell (NPC) markers in brain organoids than what was observed in fetal cortex. Additionally, the transient expression patterns of outer radial glial (oRG) genes were observed in primary cortical development, however cortical brain organoids showed continuous upregulation of oRG genes. While the authors did observe some deviations in transcriptional programs of brain organoids relative to fetal cortex development, they found only fluctuations in the expression of glycolytic and ER-stress genes in both brain organoids and primary cortical development with no drastic increase at advanced time-points (Cheroni et al., 2022). Uzquiano et al. (2022) corroborated these findings using a longitudinal single-cell atlas with eight timepoints across 6 months of brain organoid development and spatial transcriptomics of 10 organoids at one, two, and 3 months profiled by Slide-seqV2. The authors found that, during the first one to 6 months of organoid development, most differentiation transitions and cell type specification signatures matched those observed *in vivo*. Organoid NPCs, mostly apical radial glia (aRG), restricted to the center of organoids demonstrated an enrichment of glycolysis and hypoxia genes comparable to those observed in fetal tissue. This research suggests that the specification of most cortical cell types in organoids are unaffected by diverging metabolic states (Uzquiano et al., 2022). Interestingly, studies of long-term human cortical organoid maturation revealed consistent levels of stress pathway genes rather than compounding cellular stress over time. The researchers also found that some postnatal maturation features were observed between days 250 and 300 of *in vitro* brain organoid culture, including ribonucleic acid (RNA) editing and a switch in NMDA receptor subunits characteristic of postnatal mammalian brain development (Gordon et al., 2021). Tau filaments are abnormal, intracellular aggregates of p-tau and their composition can vary greatly among neurodegenerative diseases (Goedert et al., 1992). The tau filaments of AD contain all six isoforms of tau, however only the 0N3R isoform is present during fetal brain development with the additional five isoforms expressed perinatally and the four-repeat (4R) tau constituting the majority of tau isoform expression in the adult human brain (Brion et al., 1993; Hefti et al., 2018). iPSC-derived 3D neural models embedded in Matrigel-coated alginate capsules and cultured for 25 weeks with BrainPhys media were found to express all six adult tau isoforms, although not at the proportions observed in the adult brain (Miguel et al., 2019). Additionally, ReNcell VM human neural precursor cells with lentiviral overexpression of APP and PSEN1 fAD mutations were embedded in Matrigel to form a 3D cell suspension and showed increased expression of 4R adult tau isoforms in 3D compared to 2D (Choi et al., 2014). This suggests that long term culture of AD brain organoid models, and those cultured in maturation promoting medias, could express additional tau isoforms found in AD tau filaments although optimization is required to achieve adult-like proportions of the tau isoforms. Moreover, fetal tau is hyperphosphorylated and could resemble p-tau patterns observed during AD pathology (Brion et al., 1993). While brain organoids, which model features of human brain development, may be helpful in elucidating mechanisms associated with AD related tau hyperphosphorylation, careful consideration is required when discerning a feature of fetal brain development from one related to AD pathogenesis. A brain organoid model with more postnatal and adult-like protein isoforms and neuronal features may better represent the effects of age-related brain disease etiology and pathogenesis compared to shorter-term, 2D neural cell cultures. However, maintaining organoids in sterile culture for 300 days is technically challenging and costly.

As neuronal maturation occurs and complex cellular networks begin to form during brain development, neurons develop dendritic spines and become spontaneously electrophysiologically active. Much of what we understand about neuronal network maturation is limited to rodent and other nonhuman models due to the inaccessibility of human fetal brain development. Spontaneously active neuronal networks have been detected in eight-month-old cerebral organoids using high-density, penetrating microelectrodes (Quadrato et al., 2017). Additionally, electron microscopy has revealed synapses and synaptic vesicles in cortical organoids by 4 months and these cortical organoids displayed increasing mean firing rate and burst frequency from two to 10 months measured via MEAs (Quadrato et al., 2017; Trujillo et al., 2019). The synchrony index of spontaneously active cortical organoids also increased from 2 to 8 months, plateauing from eight to 10 months. Glutamate receptor antagonists and GABA receptor agonists reduced the number of spikes indicating that both excitatory and inhibitory neurons participate in spontaneous neuronal network modulation of cortical organoids (Trujillo et al., 2019). Since it has been shown that AD organoid models demonstrate AD pathology, observing neuronal network dynamics in brain organoid models of AD could provide insight into the influences of AD pathology on neuronal network dysregulation. Researchers have generated cerebral organoid models of fAD from one hiPSC line containing a *PSEN1* mutation, one hiPSC line with an *APP* mutation, and one isogenic control hiPSC line. At 2 months, they found increased Aβ42/Aβ40 ratio and total Aβ in *PSEN1* and *APP* mutant derived cerebral organoids, respectively, and an increase in mean firing rate in both *PSEN1* and *APP* organoids compared to isogenic control derived cerebral organoids (Ghatak et al., 2019). While brain organoids do not represent the adult human brain, research suggests that brain organoids can achieve neuronal maturation to the point of functional communication of neural networks and this level of maturation might recapitulate the effects of AD specific genetic background contributing to neuronal network dysregulation.

## 3 Cellular roles to model AD in brain organoids

**Figure 2:**
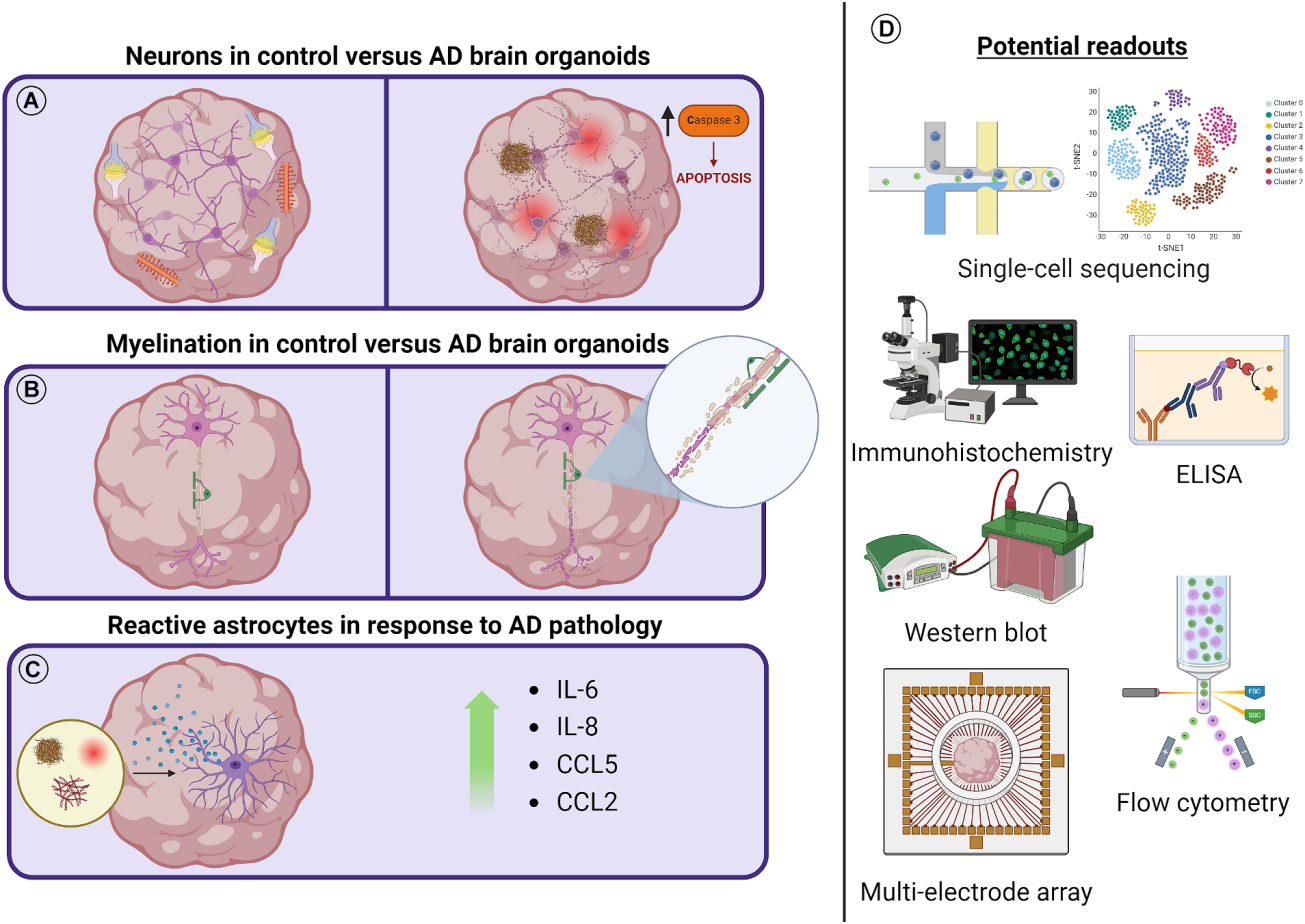
Schematic diagram outlining AD related cellular pathology and possible readouts to measure pathological effects. **(A)** As brain organoids mature, dendritic spines begin to form in neurons and functional synapses spontaneously fire. Aβ aggregates, increased p-tau and NFTs, decreased PSD95, and increased cleaved caspase-3 have all been observed in brain organoid models of AD. **(B)** Myelinating oligodendrocyte populations have been identified in unguided brain organoids after long-term culture or as early as 50 days using directed brain organoid protocols. It might be possible to observe the demyelination of axons and transcriptional or protein level regulation of this process in AD brain organoid models. **(C)** Astrocytes have been shown to increase inflammatory cytokine secretion in the presence of AD pathology. The reactivity of subpopulations of astrocytes in either unguided or astrocyte enriched brain organoid protocols can be studied in the presence of AD pathology. **(D)** Tools to observe cellular responses to AD pathology and the dysregulation of cellular processes in AD brain organoid models include scRNA-seq, ELISA, Western blot, flow cytometry, and multielectrode array technologies. Not to scale.

Historically, neuronal cells have been the focus of understanding neurodegenerative diseases, such as AD. However, roughly half of the cells in a typical brain are glial cells and about 10 to 15% of glial cells are microglia, 20 to 40% are astrocytes, and approximately 75% are oligodendrocytes (Pelvig et al., 2008). While understanding subtypes of neurons that show exceptional vulnerability to AD pathology is informative in elucidating disease progression, glial cells have critical roles in healthy brain homeostasis and could be dysregulated causing AD pathogenesis. In the brain, APOE is predominantly expressed by glial cells, and there is increasing evidence that *APOE4* glial cells exhibit detrimental differences in comparison to their isogenic *APOE3* counterparts (TCW et al., 2022). Available brain organoid protocols generally produce astrocytes, albeit amounts are often less than what is observed *in vivo* and maturation of astrocytes begins late, around 77 days of culture (Verkerke et al., 2024). Brain organoids have been developed with an enrichment of astrocytes, microglia, and oligodendrocytes through the exogenous application of platelet-derived growth factor-AA (PDGF-AA) and additional factors (Madhavan et al., 2018; Wang et al., 2024) or with the overexpression of the myeloid inducing transcription factor PU.1 to establish microglia-like cells in cortical organoids (Cakir et al., 2022). Moreover, microglia integrated into brain organoids at the erythrocyte myeloid progenitor cell (EMP) stage take on a more homeostatic morphology and transcriptional state as they convert to microglia in the brain organoid environment (Schafer et al., 2023). Although the presence of multiple cell types at more physiological proportions likely improves the representation of AD pathogenesis in brain organoids, it is challenging to attribute an observed pathological feature to a specific cell type using these complex systems. Moreover, inter- and intra- batch variability observed in brain organoids emphasizes the need to normalize for cell type composition during analyses (Hernández et al., 2022). Here, we discuss potential cell type specific contributions to AD-etiology and pathogenesis and what roles these cell types might play in brain organoid models of AD (Figure 2).

### 3.1 Neurons

Postmortem examinations of patients with AD have identified decreases in neuron number and tissue volume of the EC, layer II, and CA1 (Price et al., 2001). Despite the substantial loss of neurons, the direct cause of neuronal death and synapse loss in AD is unclear. Soluble Aβ oligomers, hyperphosphorylated tau, neuroinflammation, and age-related mitochondrial damage have all been implicated, yet there is no consensus on how the interplay of these pathological features might lead to late-onset neurodegeneration (Knott et al., 2008; Mangalmurti and Lukens, 2022). Research has suggested that soluble Aβ oligomers are major contributors in AD pathogenesis and causative relationships between altered APP processing and increased p-tau have been investigated (Israel et al., 2012). Even so, little is understood regarding the selective vulnerability of subpopulations of neurons. While selectively vulnerable networks have been characterized in AD, characterization of selectively vulnerable neurons and mechanisms underlying the vulnerability of these neurons remain elusive (Seeley et al., 2009; Schöll et al., 2016; Leng et al., 2021). Single-nucleus RNA sequencing (snRNA-seq) of postmortem brain tissue from patients with no, early, and late stages of neurofibrillary pathology in EC and SFG supported previous findings that excitatory neurons display a greater susceptibility than inhibitory neurons to AD pathology. More specifically, a population of RAR-related orphan receptor B (RORB) expressing excitatory neurons with exceptional vulnerability in AD was identified. IHC analysis showed a decrease in RORB^+^ excitatory neurons associated with early disease stages and an increase in p-tau (CP13) in RORB^+^ neurons when compared to RORB^-^ excitatory neurons (Leng et al., 2021). Brain organoids have been developed containing both excitatory and inhibitory neurons. Protocols to culture sliced cortical organoids with reduced necrosis demonstrated RORB^+^ excitatory neurons with laminar expression patterns (Quadrato et al., 2017; Qian et al., 2020). Although the maturation state of neurons in brain organoids might not represent that of an aged adult, brain organoids develop heterogenous populations of neurons and could be used to study vulnerable subtypes of neurons implicated in AD.

### 3.2 Astrocytes

Astrocytes are essential for maintaining healthy neuronal network communication by supporting the formation and function of synapses through homeostatic regulation and synaptic pruning (Diniz et al., 2012; Chung et al., 2013; Clarke and Barres, 2013). Astrocyte activation is an early response to Aβ plaque and p-tau accumulation that can upregulate nuclear factor kappa B (NFκB) signaling and increase the production of proinflammatory cytokines, including interleukin 6 (IL-6), interleukin 8 (IL-8), and C-C motif chemokine ligand 5 (CCL5) (Carter et al., 2012; Heneka et al., 2014; Lian et al., 2015; Liddelow et al., 2017; Hyvärinen et al., 2019). However, it is unclear if the chronic activation of astrocytes is the cause of or a response to Aβ deposition and NFT accumulation which may lead to the dysregulation of neuronal networks and the death of neurons in sAD. It is thought that Aβ induced astrocyte NFκB activation results in the release of complement component 3 (C3) leading to calcium dysregulation at neuronal synapses and aberrant dendritic morphology (Mattson, 2007; Lian et al., 2015). Furthermore, research suggests that an Aβ induced upregulation of inflammatory cytokines could contribute to a dysregulation of neuronal networks in sAD. Injections of IL-1β have been shown to induce febrile seizures in mice, likely through the dysregulation of Glutamatergic and GABAergic signaling, while the treatment of cultured hippocampal neurons with TNF-α increased AMPA receptors causing a greater frequency of excitatory postsynaptic currents (Knutson et al., 2002; Dubé et al., 2005; Mosili et al., 2020). Additionally, MCP-1 applied to rat substantia nigra slices increased cell excitability and dopamine release of dopaminergic neurons (Guyon et al., 2009). Chemokines could modulate neuronal activity in several neuronal subtypes and through various mechanisms, possibly by increasing calcium transients, governing the release of neurotransmitters, or effecting voltage-dependent channels (Guyon and Nahon, 2007; Rostène et al., 2007).

Much of what is known about possible astrocyte reactivity and dysfunction in AD has been investigated in AD rodent models which represent AD pathology yet lack the response of astrocytes with a human specific sAD genetic background. Although, a study using snRNA-seq to analyze postmortem EC samples of AD patients identified AD associated astrocyte subclusters with one such cluster demonstrating an enrichment for ribosomal, mitochondrial, neuron differentiation, and heat shock responses while another cluster showed downregulation of these processes and an increase in TGF-β signaling, immune responses, and C3 (Grubman et al., 2019). Additionally, Mathys and colleagues performed scRNA-seq on prefrontal cortex (PFC) with high or low levels of Aβ and found an astrocyte population associated with AD that expressed GLUL and the AD risk factor CLU, demonstrated to be upregulated in reactive astrocytes (Mathys et al., 2019). More recently, Leng et al. (2021) employed snRNA-seq and located astrocyte subpopulations in the EC and SFG corresponding to reactive astrocytes. They observed astrocyte subpopulations expressing high levels of GFAP and these GFAP high populations consistently expressed lower levels of genes associated with glutamate/GABA homeostasis and synaptic adhesion/maintenance (Leng et al., 2021). While analyses of postmortem brain tissues using snRNA-seq provides a snapshot of human AD pathology, it lacks the ability to probe dynamic cellular mechanisms contributing to AD pathogenesis and loses some spatial information that might correlate transcriptional changes with cellular proximity to pathological features of AD.

Recent protocols describing the enrichment and accelerated induction of astrocytes in cortical organoids could fill in the gaps between nonhuman animal models and postmortem tissue studies. An accelerated astrocyte induction cortical organoid protocol achieved functional astrocytes by day 45 and advanced neuronal maturation. Applying the described protocol, the authors generated chimeric organoids with *APOE* genotypes varying by cell type to investigate neuronal lipid droplet, cholesterol, and p-tau levels. The authors found that both astrocytic and neuronal *APOE4* genotypes were required to increase p-tau levels although Aβ levels were increased with astrocytic *APOE4* alone (Huang et al., 2022). An additional study induced a gliogenic switch during early cortical organoid development and achieved approximately 25 to 31% astrocytes by about day 63. The researchers identified a reactive subpopulation of astrocytes that rapidly activated upon cytokine stimulation. Implementing a similar assay in AD derived organoids might reveal more or exacerbated AD astrocyte responses to proinflammatory stimuli. These studies allow for more physiological amounts of human astrocytes in AD organoids to better recapitulate AD pathogenesis (Wang et al., 2024).

### 3.3 Oligodendrocytes

Oligodendrocytes are gaining increasing attention for their implications in AD, as their primary role is to generate myelin sheaths that act as electrical insulation of axons and wrap the axons of multiple neurons together forming white matter (WM) of the central nervous system (CNS). WM abnormalities and demyelination are well documented in early AD progression (Desai et al., 2010; Radanovic et al., 2013; Lee S. et al., 2016). Oligodendrocytes are also vital in maintaining neuron homeostasis by mediating inflammation and providing metabolic support (Fünfschilling et al., 2012; Saab et al., 2016). Like astrocytes and neurons, little is known about subtypes of oligodendrocytes that may arise during AD pathogenesis and even less is known about the molecular changes that emerge in cells related to proximity of AD pathological features. snRNA-seq of postmortem EC and SFG revealed that oligodendrocytes exhibit a discernable transcriptional response in AD with *LINGO1* as a top differentially expressed gene in oligodendrocytes and excitatory neurons. *LINGO1* is a negative regulator of myelination, neuronal survival, axonal integrity, and oligodendrocyte differentiation (Grubman et al., 2019; Mathys et al., 2019; Leng et al., 2021). Additionally, snRNA-seq and spatial transcriptomics (ST) of postmortem tissues recognized *CRYAB*, a myelin related gene, as dysregulated in oligodendrocytes within the proximity of amyloid deposition (Chen et al., 2020; Leng et al., 2021). ST studies in a mouse model of AD further identified a gene co-expression network expressed mainly in oligodendrocytes that was enriched for genes related to myelination. This gene expression network was activated with mild amyloid stress yet decreased in the presence of high amyloid accumulation. When human orthologs within the identified mouse gene network were compared to human control and AD postmortem brain samples using ST, they found that many of the genes were also significantly changed in late-stage, human AD samples but not all the genes of the network were dysregulated or enriched only in oligodendrocytes (Chen et al., 2020). ST analysis of brain organoids could advance our understanding of the cellular subtype specific responses to AD pathology and cellular mechanisms involved in AD etiology and pathogenesis. Cells with oligodendrocyte identity have been reported in unguided brain organoid protocols after long-term culture (Tanaka et al., 2020). The implementation of patterning factors, including PDGF-AA, insulin-like growth factor 1 (IGF-1), and thyroid hormone (T3) during cortical organoid generation have increased myelinating oligodendrocyte populations in brain organoids as early as day 50, however myelinated organoids have yet to be used to investigate the role of oligodendrocytes in AD etiology and progression (Madhavan et al., 2018; Marton et al., 2019; Shaker et al., 2021).

### 3.4 Microglia

Microglia are specialized macrophages of the CNS that clear debris, pathogens, and neuronal synapses. Under homeostatic conditions, microglia provide support to neurons, whereas under pathological conditions, microglia often prune synapses excessively, increase inflammation, and reduce support to neurons (Li and Barres, 2018). During AD pathological challenge, microglia are thought to contribute to neuroinflammation by increasing the secretion of pro-inflammatory cytokines (IL-1β and TNFR1) and chemokines, while reducing the amount of neurotrophic factors produced (DiSabato et al., 2016). Acute activation of microglia can promote the clearance of pathogens and toxins, although chronic activation of microglia can lead to neuronal dysfunction (Heneka et al., 2015; Calsolaro and Edison, 2016). In addition to the pro-inflammatory cytokines produced, anti-inflammatory cytokines such as IL-1 receptor antagonist, IL-4, IL-10, and IL-11 are upregulated as what could be a mechanism to prevent excessive neuroinflammation (Calsolaro and Edison, 2016). Microglia activation was thought to be triggered by AD associated Aβ deposits and dystrophic neurites, however recent genome-wide association studies (GWAS) have identified several AD risk loci in or near genes associated with microglia suggesting that microglia are influential to the etiology of AD (Verheijen and Sleegers, 2018). Furthermore, neuroinflammation may be a key player in initializing AD pathology as microglia activation has been observed prior to plaque formation in animal models of AD (Hanzel et al., 2014). This hypothesis was supported by research showing that Aβ injections alone did not induce AD amyloid pathology in primates, although injecting inflammation causing lipopolysaccharides with Aβ or injecting Aβ in animal models with chronic, systemic inflammation resulted in the formation of Aβ plaques (Philippens et al., 2016). Understanding which microglia responses are protective and which might be contributing to AD etiology could illuminate potential therapeutic targets.

Recently, Chen et al. (2023) demonstrated that APOE4/Tau mouse models experience detrimental brain atrophy compared to APOE3/Tau mice, and depleting microglia and/or T cells robustly reduced the observed atrophy (Chen et al., 2023). It is unclear if T cells or microglia are the main contributors to brain atrophy or if there is a synergistic effect. Many APOE studies in glia have utilized isogenic models to study the impact of APOE4 which neglects genetic background that could influence AD pathogenesis. Studies have demonstrated that an individual’s genetic heterogeneity can impact how APOE4 contributes to microglia dysfunction (TCW et al., 2022). However, a recent study, surveying thousands of individuals, concluded that APOE4 homozygotes represent a genetic form of AD. They revealed that the age of dementia onset for APOE4 homozygotes is earlier than APOE3 homozygotes, and that nearly all APOE4 homozygotes exhibited AD pathology and at a higher level at the age of 55 compared to APOE3 homozygotes (Fortea et al., 2024). This study emphasizes the importance of stratifying AD populations more strictly and considering separate mechanisms important in APOE4 homozygotes. Because APOE is highly expressed in glia, it would be important to parse out the role APOE4 plays in astrocytes and microglia. Microglia responses are complex, and the field has moved beyond the binary M1 and M2 microglia states. Several microglia states have now been characterized, including disease associate microglia (DAMs) and lipid droplet associate microglia (LDAMs) (Wei and Li, 2022). However, there is significant overlap among the defining features of these two states that calls for further exploration into their distinction (Haney et al., 2024). It is unclear whether DAM and LDAM states are protective responses to disease pathology, although emerging systems to study microglia can begin to address these questions. Transcriptomic studies in AD mouse models have revealed gene expression differences in microglia paralleling the progression of the disease (Keren-Shaul et al., 2017). While there is overlap between mouse and human microglia transcriptomic profiles, there is undoubtedly differences, such as an upregulation of CD58, ERAP2, GNLY and S100A12 in human microglia (Galatro et al., 2017). Additionally, human microglia have species-specific metabolic reprogramming in response to inflammation, when compared to mouse microglia (Sabogal-Guáqueta et al., 2023). The incorporation of human microglia into human brain organoids would allow for the investigation into influences of diseased microglia on healthy control organoids or vice versa to unravel human, cell type specific contributions in diseases like AD. Furthermore, characterizing the many disease-associated microglia states in proximity to AD disease pathology could shed light on the roles of these states. ST brings us closer to tackling this problem, though studies employing this method in brain organoids are limited (Norden and Godbout, 2013).

**Figure 3:**
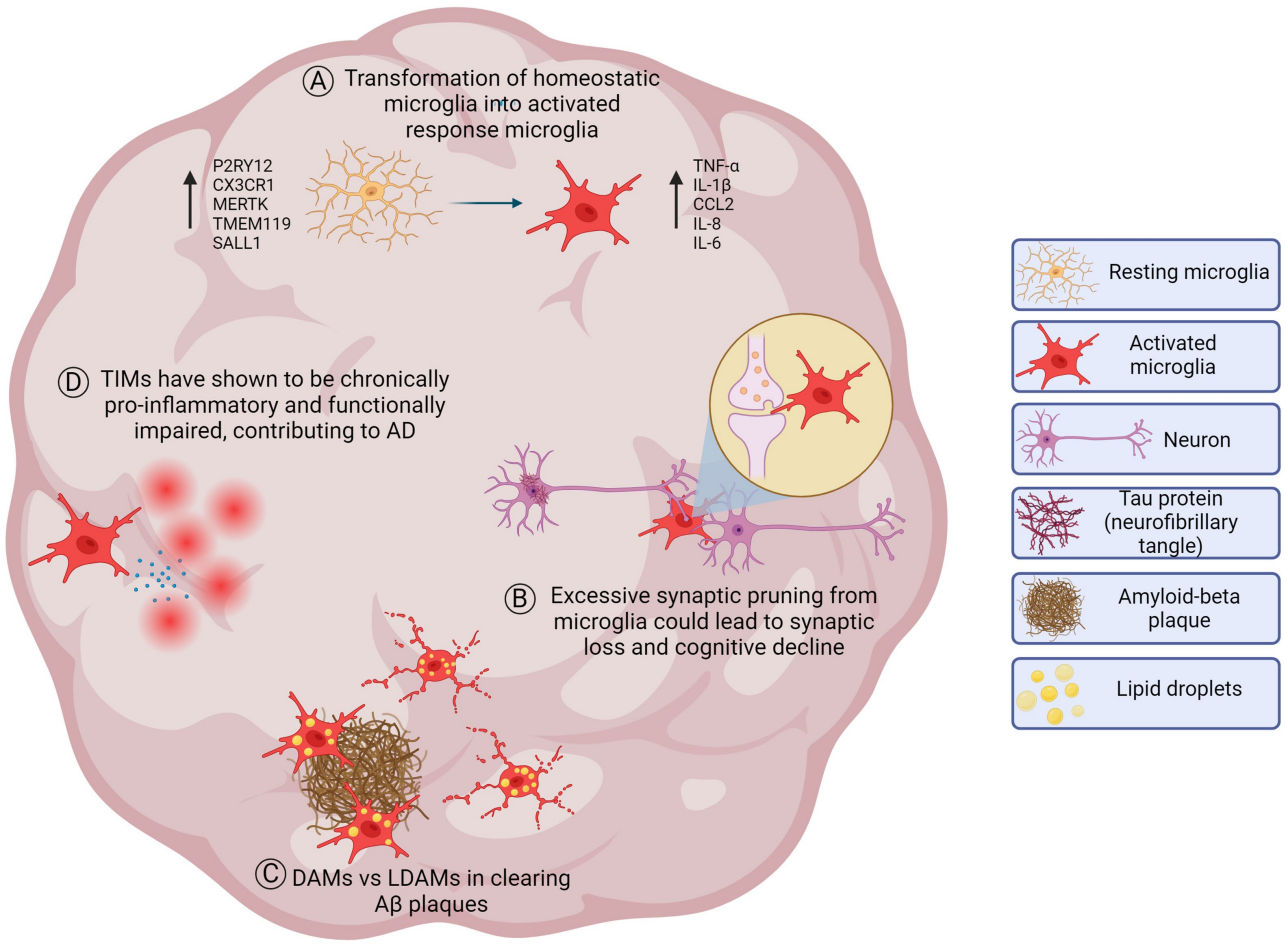
Healthy and diseased microglial processes implicated in AD which may be modeled in brain organoids. **(A)** Homeostatic microglia in a resting state in the healthy brain. Microglia participate in maintaining brain homeostasis, including synaptic pruning, surveillance of the microenvironment, and immune regulation. In response to stimuli or pathological conditions, homeostatic microglia transform into activated response microglia that have alterations in gene expression, morphology, and function. Microglial activation can promote an increase of cytokines such as tumor necrosis factor-alpha (TNF-α), interleukin-1 beta (IL-1β), chemokine ligand 2 (CCL2), interleukin-8 (IL-8) and interleukin-6 (IL-6). **(B)** Neuronal synaptic pruning. Microglia perform synaptic pruning by engulfing synaptic components to eliminate unnecessary or pathogenic synapses throughout development and disease. This process is essential for optimizing brain function. However, in AD, synaptic pruning may become dysregulated and could lead to excessive pruning leading to impairments in neuronal communication and contributing to cognitive decline. **(C)** DAMs vs LDAMs. Disease-associated microglia (DAMs) refer to microglia characterized by an upregulation of genes involved in phagocytosis, lipid metabolism, and immune response. They are thought to play a role in the clearance of amyloid beta (Aβ) plaques in AD. Lipid droplet accumulating microglia (LDAMs), on the other hand, refer to activated microglia associated with aging, late-stage disease progression, and the presence of lipid droplets. LDAMs may be senescent-like and could contribute to neurodegeneration due to dystrophic morphology and impaired phagocytic machinery reducing their ability to clear Aβ plaques. While both populations may have an increase of lipids, further investigation needs to be done to understand which lipids are accumulating (triglycerides, cholesterol esters, free cholesterol, etc.) **(D)** TIMs and inflammation. Terminally inflamed microglia (TIMs) may represent an exhausted state for inflammatory microglia that could contribute to AD risk and pathology. TIMs are senescent-like and are a terminal state for activated microglia, shown to be more inflammatory and functionally impaired. Not to scale.

Microglia have been introduced into brain organoids, allowing us to capture a more homeostatic microglia state (Figure 3). In 2D culture, microglia are prone to activation and cannot be maintained long term, even in co-culture systems (Timmerman et al., 2018). Abud et al. (2017) were the first to incorporate hiPSC-derived microglia into hiPSC 3D brain-organoids (BORGs), which contain neurons, astrocytes and oligodendrocytes. Microglia became ramified and responded to injury of the BORG, however, this study did not investigate transcriptomic changes of these microglia after incorporation into BORGs or phagocytic capacity. Further analysis would be required to understand the extent to which the microglia are maturing within the organoid. Ormel et al. (2018) modified an unguided protocol to generate microglia containing organoids, however, the amount and distribution of microglia between organoids was variable. To overcome this issue, Cakir and colleagues overexpressed transcription factor PU.1 in cerebral organoids to generate microglia-like cells in a tunable manner (Cakir et al., 2022). They went on to engraft these brain organoids into immunocompromised mouse brains to introduce vasculature into the brain organoid model. Still, limitations included variable distribution of microglia-like cells in the organoids, and limited conversion of some PU.1 expressing cells to mature microglia resulted in heterogenous microglia precursor populations. Additionally, this method requires the establishment of stable lines for each iPSC line of interest which is a timely effort when using large cohorts. Schafer et al. (2023) developed a different approach and found that infiltrating EMPs, generated using protocols from Abud et al. (2017), into forebrain organoids and then subsequently transplanting them to immunodeficient mice, allowed the EMPs to convert to microglia, taking on a more homeostatic morphology and transcriptional state when compared to *in vitro* microglia (Abud et al. 2017; Schafer et al., 2023). The introduction of EMPs into the organoids at 5 weeks mimicked the invasion of yolk sac derived EMPs into the fetal brain at this gestational period. Transplanting these microglia-enriched organoids into the cortex of immunocompromised mice advanced microglial maturation. The microglia became more ramified and demonstrated increased expression of P2RY12 and TMEM119, features of homeostasis, when compared to *in vitro* microglia. However, it is unclear how the mouse host cells might be interacting and influencing the implanted brain organoid beyond increasing oxygen and nutrient penetration and delivery. Most recently, Park et al. (2023) infiltrated iPSC-derived primitive macrophages (iMacs) into cerebral organoids. After infiltration, iMacs expressed microglia markers such as P2RY12, CX3CR1, and SALL1 at a higher level compared to *in vitro* iMacs (Park et al., 2023). Notably, these iMacs (called iMicro once infiltrated) did not express microglia marker TMEM119, which urges a comparison to existing microglia protocols to ensure these iMacs do fully convert to microglia. This study concluded that iMicro are enriched with PLIN2+ lipid droplet that export cholesterol, which were taken up by neural progenitor cells (NPCs). They found that organoids with iMicros were reduced in size and cell number, due to reduced proliferation of NPCs, which they suggest is a consequence of iMicro protecting against excessive growth. This study highlights the importance of cholesterol transport in development, potentially via APOE lipoproteins. While the direct study of microglia in AD organoids is limited, Haney et al. (2024) recently showed that conditioned media from lipid droplet high APOE44 microglia, compared to lipid droplet low, induced Tau phosphorylation and apoptosis in neurons. Together, this prompts the field to investigate the role microglia plays in cholesterol transport to other cell types and how this pathway is altered in AD, which could be further elucidated with microglia-rich organoids.

## 4 Challenges and future directions

Possibly the greatest challenge of implementing brain organoid technologies for the investigation of late-onset diseases like AD is the fact that brain organoids are derived from embryonic-like cells. As brain organoids develop, they recapitulate features of early brain development and are deficient in some *in vivo* systems which might be limiting their capacity for cellular maturation. One important feature that is lacking in brain organoid models is vasculature. While the lack of vasculature might enhance some AD-related pathological features in brain organoids, such as Aβ aggregation, it limits oxygen and nutrient diffusion into the organoid causing cellular stress and necrosis. Aging has been defined as the time dependent accumulation of cellular damage (Kennedy et al., 2014). While it is unrealistic to model every aging component influencing age-related diseases using brain organoids, it could be feasible that some phenotypic features of age-related brain diseases are driven by genetic background or cellular stress and may be apparent prior to symptom onset. Early phenotypic features of AD might be revealed in brain organoid models. Additionally, age-related signatures, including epigenetic aging markers, of a patient or non-affected donor can be retained via the transdifferentiation of somatic cells to neural cell types (Gatto et al., 2021; Mertens et al., 2021; Herdy et al., 2022). The organization of transdifferentiated neural cell types into 3D cellular models could reveal the epigenetic and environmental contributions of multiple cell types to AD pathogenesis.

Recent advances in 3D cell biology, materials science, and bioengineering have produced models of increasing complexity with a systems wide approach. Assembloids consisting of fused brain organoids with various brain region specific identities have bene established and suggest that increased brain region specification of brain organoids models could allow for the study of dysregulated brain region connectivity in AD that could be implicated in cognitive impairment (Marton and Pașca, 2020). Furthermore, the BBB protects the CNS from influencing blood-borne materials and, although some changes to the BBB may occur during healthy aging, its disruption is thought to cause neuronal dysfunction contributing to AD (Banks et al., 2021). A BBB-like system might be incorporated into brain organoids to investigate the potential roles of endothelial cells (ECs), pericytes, microglia, astrocytes, and neurons contributing to vascular dysfunction in AD. Currently, BBB spheroids have been established through the spontaneous aggregation of primary human astrocytes, human brain vascular pericytes, and either primary brain microvascular ECs or immortalized human cerebral microvascular EC line D3. These BBB spheroids demonstrate tight junctions, efflux pumps, and transporters, albeit they lack the influence of microglia and neurons (Cho et al., 2017). Additionally, organs-on-chips technology or microfluidic-chip-based systems containing compartments where cells can be co-cultured in either 2D or 3D architectures, recapitulating microenvironments of human tissues like BBB, might be employed (Li et al., 2020). The gut-brain axis has been identified as an important relationship in homeostasis and pathophysiology. Organs-on-chips technology and iPSC derived gastrointestinal organoids might be combined with brain organoids to mimic the microbiome-gut-brain axis while maintaining patient specific genetic background and influence (Sato et al., 2011; Fujii et al., 2018; Min et al., 2020). Several potential AD drugs which showed promise during animal trials failed during phase III clinical trials (Doody et al., 2014; Salloway et al., 2014). Transwell models, microfluidic systems, or organoid BBB models could one day allow for high-throughput screening of compounds for drug discovery but are currently challenging to scale up (Urich et al., 2013; Hajal et al., 2022). Scaling up human brain organoid technology, from brain organoid generation to systematic, high throughput drug screening and analyses, may accelerate AD drug development (Park et al., 2021). Brain organoids provide access to some AD pathology, cellular interactions of multiple cell types implicated in AD, human specific genetic background influencing sAD etiology and pathogenesis, and AD-related cellular mechanisms that might be perturbed by extrinsic, modulating factors, all of which could reveal potential therapeutic targets. Even though human based cellular models considering multiple systems could advance our understanding of age-related brain diseases and drug development, they will not replace nonhuman animal models or postmortem human brain tissue in elucidating certain aspects of age-related brain diseases. Brain organoids are far from modeling the implications of cellular function on behavior and the effects of AD pathogenesis on aged, adult brain architecture.

## Author contributions

SF: Conceptualization, Writing – original draft, Writing – review & editing. JR: Conceptualization, Writing – original draft. JP: Conceptualization, Visualization, Writing – original draft. NH: Conceptualization, Visualization, Writing – original draft. MM: Conceptualization, Supervision, Writing – review & editing. FG: Conceptualization, Supervision, Writing – review & editing.
